# Global ecological predictors of the soil priming effect

**DOI:** 10.1038/s41467-019-11472-7

**Published:** 2019-08-02

**Authors:** Felipe Bastida, Carlos García, Noah Fierer, David J. Eldridge, Matthew A. Bowker, Sebastián Abades, Fernando D. Alfaro, Asmeret Asefaw Berhe, Nick A. Cutler, Antonio Gallardo, Laura García-Velázquez, Stephen C. Hart, Patrick E. Hayes, Teresa Hernández, Zeng-Yei Hseu, Nico Jehmlich, Martin Kirchmair, Hans Lambers, Sigrid Neuhauser, Víctor M. Peña-Ramírez, Cecilia A. Pérez, Sasha C. Reed, Fernanda Santos, Christina Siebe, Benjamin W. Sullivan, Pankaj Trivedi, Alfonso Vera, Mark A. Williams, José Luis Moreno, Manuel Delgado-Baquerizo

**Affiliations:** 1 CEBAS-CSIC, Department of Soil and Water Conservation, Campus Universitario de Espinardo, 30100 Murcia, Spain; 2 Cooperative Institute for Research in Environmental Sciences, University of Colorado, Boulder, CO 80309 USA; 3 Department of Ecology and Evolutionary Biology, University of Colorado, Boulder, CO 80309 USA; 4 Centre for Ecosystem Science, School of Biological, Earth and Environmental Sciences, University of New South Wales, Sydney, NSW 2052 Australia; 5 School of Forestry, Northern Arizona University, 200 E. Pine Knoll Dr., Box 15018, Flagstaff, AZ 86011 USA; 6 GEMA Center for Genomics, Ecology & Environment, Universidad Mayor, Camino La Piramide 5750, Huechuraba, Santiago 8580745 Chile; 7 Instituto de Ecología and Biodiversidad (IEB), Casilla, Santiago, 653 Chile; 8 Centro de Biodiversidad y Genética (CBG), Facultad de Ciencias y Tecnología, Universidad Mayor de San Simón, Sucre y Parque La Torre, Cochabamba, 538 Bolivia; 9 Department of Life and Environmental Sciences, and Sierra Nevada Research Institute University of California, Merced, CA 95343 USA; 10 School of Geography, Politics and Sociology, Newcastle University, Newcastle, NE1 7RU UK; 11 Departamento de Sistemas Físicos, Químicos y Naturales. Universidad Pablo de Olavide, 41013 Sevilla, Spain; 12 School of Biological Sciences, The University of Western Australia, Perth, WA 6009 Australia; 13 Centre for Microscopy, Characterisation and Analysis, The University of Western Australia, Perth, WA 6009 Australia; 14 Crop, Livestock and Environment Division, Japan International Research Centre for Agricultural Sciences, Tsukuba, Ibaraki 305-8656 Japan; 15 Department of Agricultural Chemistry, National Taiwan University, Taipei, 10617 Taiwan; 16 Helmholtz-Centre for Environmental Research – UFZ, Department of Molecular Systems Biology, Permoserstrasse, 15, 04318 Leipzig, Germany; 17 Institute of Microbiology, University of Innsbruck, Technikerstrasse 25, A-6020 Innsbruck, Austria; 18 Instituto de Geología, Universidad Nacional Autónoma de México, Ciudad Universitaria, México D.F., CP 04510 Mexico; 19 Instituto de Ecología y Biodiversidad, Las Palmeras, Santiago, 3425 Chile; 20 U.S. Geological Survey, Southwest Biological Science Center, Moab, UT 84532 USA; 21 Department of Natural Resources and Environmental Science & The Global Water Center, University of Nevada, Reno, 1664 N. Virginia Street, Reno, NV 89557 USA; 22 Department of Bioagricultural Sciences and Pest Management, Colorado State University, Fort Collins, CO 80523 USA; 23 School of Plant and Environmental Sciences, Virginia Tech, Blacksburg, VA 24061 USA; 24 Departamento de Biología y Geología, Física y Química Inorgánica, Escuela Superior de Ciencias Experimentales y Tecnología, Universidad Rey Juan Carlos, Calle Tulipán Sin Número, 28933 Móstoles, Spain

**Keywords:** Biodiversity, Carbon cycle

## Abstract

Identifying the global drivers of soil priming is essential to understanding C cycling in terrestrial ecosystems. We conducted a survey of soils across 86 globally-distributed locations, spanning a wide range of climates, biotic communities, and soil conditions, and evaluated the apparent soil priming effect using ^13^C-glucose labeling. Here we show that the magnitude of the positive apparent priming effect (increase in CO_2_ release through accelerated microbial biomass turnover) was negatively associated with SOC content and microbial respiration rates. Our statistical modeling suggests that apparent priming effects tend to be negative in more mesic sites associated with higher SOC contents. In contrast, a single-input of labile C causes positive apparent priming effects in more arid locations with low SOC contents. Our results provide solid evidence that SOC content plays a critical role in regulating apparent priming effects, with important implications for the improvement of C cycling models under global change scenarios.

## Abstract (web summary)

The global ecological predictors of soil priming remain unclear. Here the authors conducted a global survey of soils from 86 global locations using an isotopic approach and find that in more mesic sites with high SOC concentrations, soil priming effects are more likely to be negative.

## Introduction

Soil contains more C than the atmosphere and aboveground plant biomass combined (the top three metres of soil stores more than 2300 Pg C)^1,2^. Carbon dioxide (CO_2_) efflux from soils is one of Earth’s largest fluxes of C to the atmosphere^1^. An important part of such efflux can result from the turnover of the soil microbial biomass, which is sensitive to environmental changes^3,4^ and is estimated to contain up to 23.2 Pg C within the first top 100 cm of soil^2^. Soil priming, the change in the microbial decomposition of soil organic carbon (SOC) in response to fresh carbon (C) inputs, is a key component of global carbon C cycling^5–7^. Priming is divided into two components: apparent priming, which corresponds to change in the CO_2_ evolved from microbial biomass turnover after the input of easy-available substrates, and real priming, which corresponds to the change in CO_2_ release from soil organic matter^7,8^. These two components of priming are difficult to distinguish, but apparent priming tends to occur shortly after adding readily availably substrates (first days and weeks), while real priming takes longer^7,9^. Overall, soil priming is a complex phenomenon that is regulated by multiple mechanisms involving abiotic and biotic factors (including, but not limited to, nutrient availability, catabolism of different organic matter pools)^6,7,10,11^. Soil priming has been postulated to be a major determinant of the capacity of soils to function as sources or sinks of atmospheric CO_2_^12^. Consequently, inputs of fresh organic matter to the soil can cause an accelerated microbial biomass turnover in the short term (positive apparent priming). Alternatively, a negative apparent priming can arise from an attenuated microbial biomass turnover when labile C is added to soil^6^. Recent modelling developments suggest that soil priming is a strong candidate for inclusion in models to predict global distributions of C because of the important role of priming in determining the exchange of C between soils and the atmosphere^5,13^. However, we lack a unifying ecological context and an integrative approach to understanding soil priming effects globally, which would allow us to determine how the direction of the priming effect varies across different ecosystems and why this variation exists.

A growing body of literature has identified nutrient availability, climate, soil type, or plant and microbial attributes^14–18^ as potentially important drivers of priming^7^. For example, soil texture has been demonstrated to be an important factor controlling the soil priming effect, and plants, through the amount and composition of rhizodeposits, also play a key role in mediating priming effects^4^. Furthermore, climatic factors, such as mean annual temperature are related to soil priming effects^11^. However, in spite of the elevated amount of C within microbial biomass^2^, a comprehensive understanding of the drivers of the apparent priming effect across major biomes and gradients at the global scale is lacking. This knowledge will shed light on how environmental factors regulate the microbial biomass turnover and its contribution to CO_2_ fluxes under global change scenarios^19,20^. Moreover, a better understanding of the ecological predictors of priming will improve our ability to predict how CO_2_ fluxes might shift in response to human and global change factors that influence the quality and quantity of fresh C inputs to soil, as well as soil microbial responses^12^, such as afforestation^21^, changes in plant C allocation to soil due to the elevated levels of atmospheric CO_2_^12^, the addition of organic amendments to soil^22^, nitrogen (N) deposition^23^, warming^24^, and changes in land use^25^.

Herein, we conducted a soil survey of 86 locations across six continents, spanning multiple climates (tropical, temperate, polar, arid and continental) and ecosystem types (e.g., forest, grasslands and croplands; Supplementary Fig. 1). We aimed to identify the major global ecological predictors of the apparent soil priming effect. Apparent priming was determined using a soil incubation of 16 days coupled with ^13^C-labeled glucose. Ecological predictors included wide environmental gradients of mean annual temperature, aridity, vegetation types, plant cover, soil chemical and physical properties, and microbial attributes (microbial respiration, biomass and original soil community composition of bacteria and fungi). Moreover, information on the microbial populations potentially associated with the apparent priming effects remains limited^18^. Therefore, considering microbial attributes, as we have done here, is critical in evaluating the environmental factors predicting the apparent priming effect.

Given that SOC is widely correlated with microbial biomass^26^, we hypothesized that the effect size and the direction of the apparent priming effect is regulated by SOC content, which, in turn, is modulated by the environmental and ecological context of each soil^27,28^. Thus, we hypothesized that soils with lower SOC content, including soils from arid sites with sparse plant cover where microbial biomass is strongly limited by C^29^, will be more responsive to the inputs of labile C, ultimately stimulating microbial turnover and the resulting apparent priming-mediated CO_2_ release (positive priming)^7^. Conversely, we expected that the apparent priming effect would be negative in soils from mesic regions with greater plant cover and higher litter and root inputs to soil where microbial biomass and soil microbial respiration are less limited by the availability of C. Our cross-biome survey allows the identification of factors associated with the apparent soil priming in terrestrial ecosystems worldwide. We find that the apparent priming effect is globally ubiquitous and controlled by the SOC content, with important implications for the study of C fluxes in changing environments and for the improvement of global models of soil C dynamics.

## Results

### Patterns of apparent priming across ecosystems

**Fig. 1:**
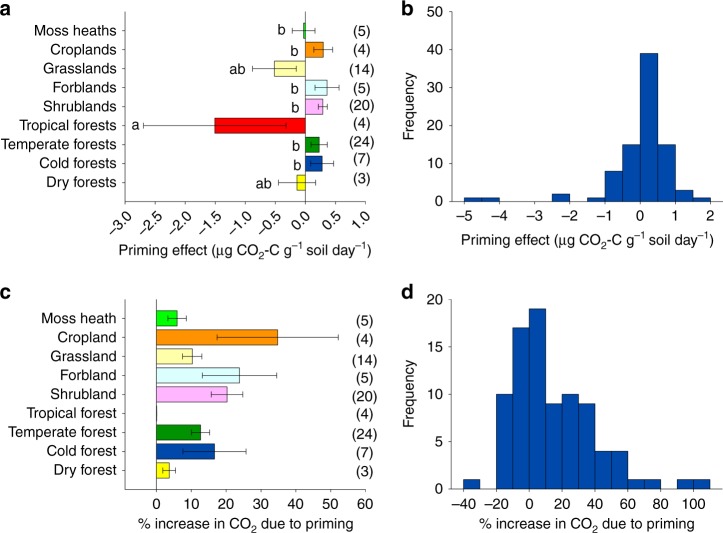
Apparent soil priming effects across globally distributed ecosystems. **a** Priming effect across major biomes. Different letters in this panel indicate significant differences among ecosystems (*p* *=* 0.007). **b** Histogram showing data distribution for the apparent priming effect. **c** Percentage of CO_2_ from apparent priming *vs*. basal soil microbial respiration rates (*p* *=* 0.50). **d** Histogram showing data distribution for the apparent priming vs. soil respiration rates. Number of samples in brackets (*n* = 86). Ecosystems are defined using major vegetation types and the Koppen classification. Number of sites is indicated in parentheses. Error bars are standard error of the mean. Source data are provided as a Source Data file

We found contrasting responses of apparent priming associated with different globally distributed ecosystem types. In some soils, a single-pulse of labile C accelerated the turnover of microbial biomass (positive apparent priming). Conversely, the addition of labile C leads to reductions in microbial turnover in other soils (negative apparent priming; Fig. 1a–b). For instance, positive apparent priming effects were associated with shrub- and forb-dominated ecosystems, croplands and cold forests (Fig. 1a). In some ecosystems (i.e. croplands, forblands and shrublands), the release of CO_2_ due to positive apparent priming represented more than 20% of the basal microbial respiration rate (Fig. 1c–d). Nevertheless, the magnitude of the positive apparent priming effect as a fraction of the total SOC pool was low (with a maximum of the 0.13% of the SOC being mineralized due to priming in cold forests; Supplementary Fig. 2), which likely corresponds to the CO_2_ released by acceleration of microbial turnover. In contrast, we found negative apparent priming effects in grasslands, and particularly, in soils with very high SOC contents (e.g., volcanic soils from Hawaii) (Source Data, Supplementary Information). These findings indicate that apparent priming responses are ecosystem dependent. In other words, the importance of the apparent priming-derived CO_2_ in soils with the highest organic C content, such as those in tropical ecosystems^30^, is typically lower than in other ecosystems supporting lower levels of soil C such as drylands and croplands^31^ (Fig. 1c–d).

### Ecological predictors of the apparent soil priming

We used structural equation modeling (SEM; a priori model in Supplementary Fig. 3) to provide integrated information on the major ecological predictors of apparent soil priming across a broad range of soil types from different ecosystems and climates (Supplementary Fig. 1; see Methods). SEM is particularly useful in large-scale studies, as it allows us to partition causal influences among multiple variables, and to separate the direct and indirect effects of the predictors included in the model^32^. Further, SEM is capable of accounting for continuous and categorical variables. Our model included important geographical and ecological factors such as climate (aridity [ARI], calculated as 1- the Aridity Index, which is negatively related to mean annual precipitation and mean annual temperature [MAT]), variables related to soil C (basal microbial respiration rates and total organic C), soil properties (Olsen phosphorus [soil P], pH, clay + silt and salinity), plant cover, dominant vegetation type (forests, shrublands, grasslands and croplands), and important microbial features such as microbial biomass (via substrate-induced respiration [SIR]), and the relative abundance of selected microbial taxa from the original microbial community in our soils (see Methods). Before conducting our SEM, we checked for potential multicollinearity among the selected ecological predictors. None of the predictors included in our SEM suffered from multicollinearity (*r* < 0.8), and therefore, multicollinearity issues were not expected in this model. Note that our SEM did not examine an explicit direct effect of aridity and mean annual temperature (MAT) on either apparent priming or respiration rates (as soils were incubated under controlled laboratory conditions). However, we included these climatic factors in our SEM to evaluate the indirect effects of climate on apparent priming via changes in SOC and plant cover, which we measured under field conditions, therefore providing an ecological context to our results.

**Fig. 2:**
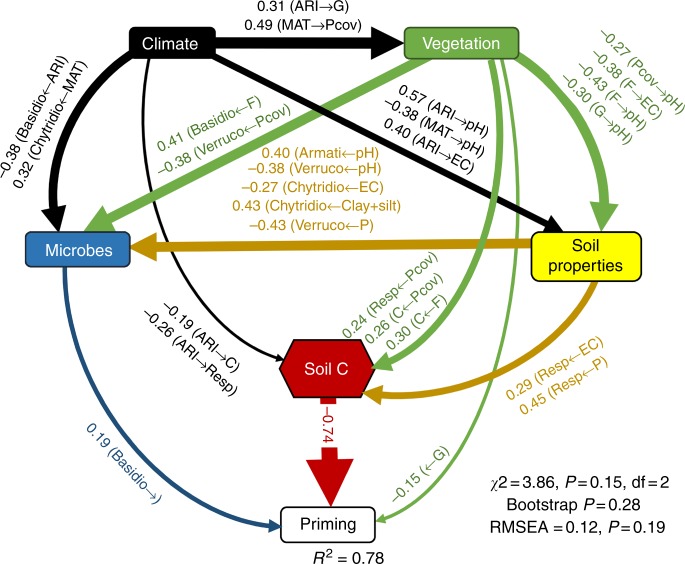
Ecological predictors of the apparent soil priming effect. Structural Equation Model (SEM) describing the effects of multiple ecological predictors on the apparent soil priming effect (*n* = 69). Numbers adjacent to arrows are indicative of the effect size (*p* < 0.05) of the relationship. *R*^2^ denotes the proportion of variance explained. Climate, soil properties and vegetation predictors are included in our models as independent observable variables; however, we group them in the same box in the model for graphical simplicity. Soil carbon (C) associated variables (soil microbial respiration and soil organic C content) are included as a composite variable in our model (hexagon). F = forest. G = Grasslands. SHR = Shrublands. C + S = Clay + silt. EC = Salinity. Resp = Basal microbial soil respiration. Basidio = % of *Basidiomycota*. Verruco = % of *Verrucomicrobia*. Armati = % of *Armatimonadetes*. Chytridio = % of *Chytridiomycota*. Pcov = % of plant cover. ARI = Aridity (i.e., 1-ARI). Locations with a higher aridity also support lower water availability). MAT = Mean annual temperature. There was a non-significant deviation of the data from the model (*χ*^2^ = 3.97, df = 2; *p* = 0.14; RMSEA *p* = 0.18)

**Fig. 3:**
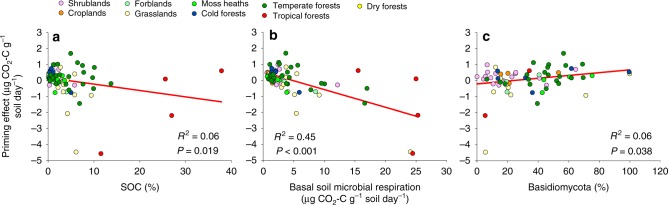
Selected relationships from SEM between the apparent priming effect and different variables. **a** Apparent priming vs soil organic C (SOC) content. **b** Apparent priming vs basal microbial respiration. **c** Apparent priming vs the relative abundance of Basidiomycota. Colour symbols represent ecosystem types. All relationships are significant (*p* < 0.05)

**Fig. 4:**
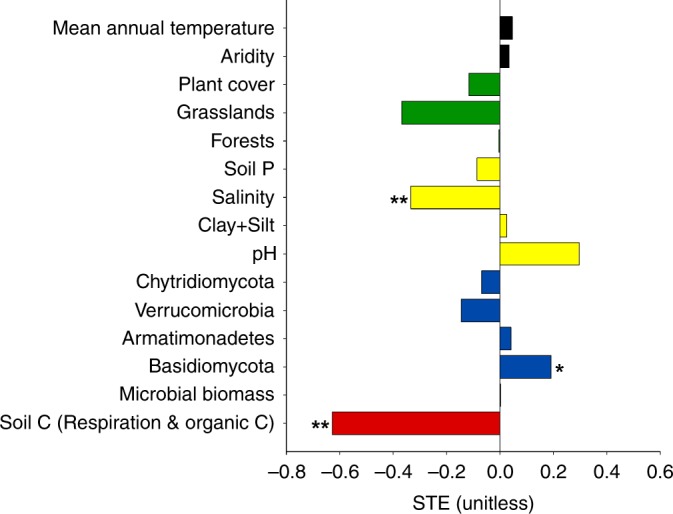
Standardized total effects (STE) from the Structural Equation Model (SEM). Sum of direct and indirect effects of multiple ecological predictors on the apparent soil priming effect (*n* = 69). Soil carbon (C) represents the sum of the standardized effect of soil organic C (SOC) and microbial respiration rates, which reflects SOC which is respired by microbes. *p < 0.05, **p < 0.01

In spite of the inherent difficulties for predicting the soil priming effect at the global scale, our SEM approach explained a large portion of the variation in the apparent priming effect worldwide (~80%; Fig. 2), and provided strong evidence that SOC content (ranging from 0.1 to 38%) and basal microbial respiration were directly and negatively associated with apparent priming effects (Figs. 2–4). Importantly, our model goodness-of-fit was strong, indicating that it represents a causal scenario consistent with the data. Strikingly, soil microbial biomass (estimated using substrate-induced respiration, SIR) was not a significant predictor of apparent priming in the wide variety of soils tested here (Fig. 2). Our results suggest that the initial content of SOC ultimately regulates the apparent soil priming effect. Soils with greater C content (therefore, less limited by C) are more likely to exhibit negative or minimal apparent priming. Importantly, the negative relationships between SOC content and apparent priming (Fig. 3a), and between basal respiration and apparent priming (Fig. 3b) were maintained even after tropical soils (the soils with the highest SOC content) were removed (SOC content vs apparent priming without tropical soils: *r* = −0.27; *p* = 0.015; basal respiration *vs* apparent priming: *r* = −0.67; *p* < 0.001).

By using amplicon sequencing approaches, we could further investigate associations between soil microbial community composition and the direction of the apparent soil priming effect. We found that soils having higher relative abundance of *Basidiomycota* and *Armatimonadetes* had higher positive apparent priming effects. Conversely, soils with higher relative abundances of *Verrucomicrobia* and *Chytridiomycota* tended to have lower or negative apparent priming effects (Fig. 3; Supplementary Table 1). However, in our SEM, only the relative abundance of *Basidiomycota* had significant direct effects on the apparent priming effect after considering multiple environmental factors simultaneously (Figs. 2–4). Further, we found 1118 phylotypes classified as *Basidiomycota* in our globally distributed soils. Among these taxa, we selected the most common (present in >10% of all locations) and conducted Random Forest analyses (as described in Delgado-Baquerizo et al.^33^) to identify the most important *Basidiomycota* taxa associated with the magnitude of the apparent priming effect across biomes. We found that taxa associated with apparent positive priming effects belonged to unidentified Agaricomycetes phylotypes (Supplementary Fig. 4).

## Discussion

Our findings indicate that the apparent soil priming effect is a globally ubiquitous phenomenon, and provide new insights into its major ecological predictors, in spite the extreme heterogeneity of soils and incubation limitations, as described below. Our work, based on short-term incubations using ^13^C-glucose, lasted 16 days and mainly reflects the patterns of the apparent priming effect that occurs within the first few days or weeks after the input of labile carbon in the soil^7,8^. These C fluxes correspond to changes in CO_2_ release as a consequence of microbial biomass turnover shortly after adding freshly available substrates^7,8^.

Our work is consistent with the results of previous studies showing that priming occurs in most soils^14,17,18^. Previous studies have demonstrated that priming is modulated by plants and rhizodeposits^17^, microbial biomass^7,34^, microbial diversity^18^ and warming^24^. Here, we decipher the ecological context that regulates the apparent priming effect by considering a large range of soils that varied in their abiotic and biotic factors. Our study suggests that a single pulse of labile C can cause contrasting responses of apparent priming (microbial turnover) across a wide gradient of soil and ecosystem types, and that initial content of SOC is a critical driver of this phenomenon. These results have implications for the prediction of C fluxes under forecasted global change and for the improvement of global C cycling models. SEM provides an ecological context for apparent priming effects across a wide range of soils. Soils with greater plant cover located in more mesic ecosystems had higher soil C contents and basal microbial respiration rates that were associated with a greater likelihood of negative apparent priming effects. A priori, the microbial community in these soils is expected to be adapted to greater C inputs from plants. In these communities, inputs of fresh substrate could be used by microbes to support growth, assimilating C in microbial biomass and thus limiting the release of CO_2_ to the atmosphere, explaining the negative apparent priming effect in these soils.

Conversely, our results suggest that positive apparent priming is likely greater in soils under drier climates (i.e. shrublands) and with land use (e.g., croplands) with low SOC contents^28,31^. A previous study using an herbaceous savannah soil, also revealed that positive priming effects were more likely to be observed in nutrient-limited soils^16^. The microbial community of these soils is likely poorly adapted to the input of fresh-organic C and might respond with an intense turnover to glucose addition. Further, the distribution of aerobic and anaerobic populations can vary in soil depending on the amount of soil aggregates^35^. Soils from arid locations, with a low content of organic C and likely lower amounts of macroaggregates, could contain a relatively higher content of aerobic bacteria than can be associated with positive priming. Conversely, tropical soils with a high organic C content and high moisture could contain a proportionally higher abundance of facultative or anaerobic microorganisms that could be associated with negative apparent priming. Indeed, a reduction of soil organic C mineralization (negative priming effect) has been found in anaerobic conditions^36^. An additional explanation could be that soils under arid or semiarid climates are not adapted to the soil water content utilized in the incubation (50% of the water-holding capacity) and microbial turnover could be stimulated in such conditions, contributing to the release of CO_2_^37^. These findings have important implications for the future of C cycling in drylands, which are predicted to expand by up to 23% during this century^38^, and cropping areas, which are expected to increase to support a growing human population.

Previous studies have suggested that the total content of N and phosphorus (P), as well as C:N and N:P ratios of the soil organic matter (SOM), play a major role in the direction of priming^18^. For instance, Chen et al.^39^ found that the interactions between C and N availability influenced the extent of the priming effects. Moreover, other authors have found that priming can be more significant in N- and P-limited soils because microbes need to mine the SOM for such elements in nutrient poor environments^9,16,40^. In contrast, recent novel dual isotope approaches (^13^C- and P-^18^O tracers) have revealed a stronger priming effect in soils with larger P contents than in soils with smaller P contents^41^. In our study, which centered on apparent priming effects, soil N content was highly correlated with SOC content (*r* = 0.88; *p* < 0.001), and was therefore not included in our statistical modeling to avoid multicollinearity. Further, available soil P (Olsen P) content did not correlate significantly with the apparent priming effect (*r* = −0.27; *p* = 0.81). In this respect, our study suggests that, across broad gradients in soil P availability, available soil P might have a relatively small role in driving the microbial turnover responsible on the apparent priming effects. Moreover, soil elemental stoichiometry, not included in our a priori model, was not correlated with the apparent priming (total N: available soil P: *r* = −0.07; *p* = 0.533 and total organic C: total N: *r* = −0.15; *p* = 0.181). Similarly, physical factors such as soil texture, which has also been proposed as a factor regulating soil priming effects^34^, was not a significant factor across the broad range of soils tested here. Other soil properties, such as pH, available soil P content and salinity did not show any direct effect on the apparent soil priming, but these factors indirectly affected soil microbes, and salinity had a total negative significant effect on priming^42,43^.

*Basidiomycota* are dominant and widely-distributed fungi^44^ that play important roles as decomposers of plant-derived organic matter^45^. Further, *Basidiomycota* have been reported to become active through the utilization of glucose and then to change their substrate preference to native SOC compounds, which also include microbial necromass as a fundamental component^46,47^, once glucose or other labile C compounds are depleted^11^. Several studies have shown that Gram-negative bacteria generally outcompete fungi for glucose during the initial stages^14,48^, but that some fungi, including Basidiomycota, can later feed on bacterial biomass, with some recent studies demonstrating short-term foodwebs fueled by glucose in soil^49,50^. Thus, the feeding by Basidiomycota on bacterial biomass (and necromass) could be a feasible mechanism for the apparent priming effect observed here. Further, we highlight the fact that soil was sieved through 2 mm prior to incubation (see Methods), and it might be possible that *Basidiomycota* hyphae were fragmented, although their DNA can be still present in soil as relic DNA^51^. The subsequent microbial decomposition of fungal hyphae fragments during the incubation could contribute to the apparent positive priming in soils with greater abundance of *Basidiomycota*. Alternatively, the decomposition of basidiomycotal mycelia through several Gram-negative populations has recently been demonstrated^52^. Moreover, basidiomycotal spores and fragments of hyphae (diameter of 4–6 µm vs. sieving at 2000 µm) can resist sieving and develop during the incubation, contributing to the observed priming results.

Nevertheless, we acknowledge some limitations of our study. First, the size of the incubation (1 g of soil) did not sufficiently account for the presence of macroaggregates. However, it is known that soil aggregates are critical for C sequestration^53,54^ and that aggregate disruption through sieving can influence priming effect patterns^55^. Given their connection with C sequestration, further models of priming should also consider the content of aggregates. Second, incubation conditions in our study differed from those likely experienced in the field (i.e. different temperature and soil water content). Consequently, our results should be interpreted as potential patterns of apparent priming. Even if our experimental incubation did not fully replicate in situ conditions, such experimental data can be useful for evaluating assumptions underlying microbially-explicit soil biogeochemical models, and help to identify how microbial processes and edaphic factors can drive apparent priming at the global scale.

Together, our work provides a comprehensive perspective on the ecological predictors underpinning the direction of apparent priming effects across a wide range of soils from different ecosystems and climates. The identification of the major ecological predictors of apparent soil priming across such a broad spatial scale and the consistency of variation for this phenomenon in an ecosystem-dependent manner, significantly improves our understanding of the potential turnover of microbial biomass and its contribution to CO_2_ fluxes in soil. In agreement with the suggested hypothesis, our findings highlight the fact that the apparent priming effect is globally ubiquitous and controlled by the SOC content. Importantly, we place priming within an ecological context, showing that apparent soil priming is positive (accelerated microbial biomass turnover after glucose input) in soils with high aridity and relative abundance of *Basidiomycota*, and low plant cover, SOC content and basal microbial respiration rates. Further, our results indicate that salinity is an important negative driver of the apparent soil priming effect worldwide. These findings help identify the predictors of apparent soil priming in terrestrial ecosystems, with important implications for the study of C fluxes under forecasted climate change and for the improvement of global models of soil C dynamics. Further studies should extend the mechanistic understanding of priming, including more functional aspects of the dynamics of the microbial community and their role in soil priming through the use of approaches based on stable isotope labelling, and the chemical composition of organic matter, not only in terrestrial ecosystems, but also in aquatic ecosystems where priming effects also have been demonstrated to be important^10^.

## Methods

### Soil sampling

Soil and vegetation data were collected between 2016 and 2017 from 86 locations in six continents (Supplementary Fig. 1). These locations include a wide range of globally distributed soil, vegetation (including grasslands, shrublands, forests and croplands) and climate (tropical, temperate, continental, polar, and arid) types. Sampling was designed to obtain wide gradients of edaphic characteristics across soil formation stages while constraining climate^56,57^. Mean annual temperature ranged between −1.8 and 21.6 °C, and Aridity Index between 0.08 and 4.33. Soils utilized in this study belong to a global collaborative network of soil chronosequences^58^. Field surveys were conducted according to a standardized sampling protocol^59^. In each location, we surveyed a 50 m × 50 m plot. Three parallel transects of the same length, spaced 25 m apart were added. The cover of perennial vegetation was measured in each transect using the line-intercept method^59^. Plant cover ranged between 0 and 100%. One composite topsoil (five 0–10 cm soil cores) sample was collected under the dominant ecosystem features across our plots (e.g., trees, shrubs, grasses, croplands). Following field sampling, soils were sieved (<2 mm) and frozen at −20 °C.

### Soil chemical and physical analyses

For all soil samples, we measured electrical conductivity, pH, texture, SOC content and available P (Olsen P) content. Soil properties were determined using standardized protocols^59^. Soil pH was measured in all the soil samples with a pH meter, in a 1: 2.5 mass: volume soil and water suspension. Soil texture (percentage of fine fractions: clay + silt) was determined according to Kettler et al.^60^. Total N was obtained using a CN analyzer (LECO CHN628 Series, LECO Corporation, St Joseph, MI USA). The content of Olsen P was determined from bicarbonate extracts using colorimetric analyses as explained in Olsen and Sommers (1982)^61^. SOC content ranged between 0.1 and 38%, available P between 0.5 to 72 mg P kg^−1^ soil, pH between 3.8 to 9.1 and the percentage of clay + silt varied between 0.3 and 86%, respectively.

### Experimental incubation

As sugars are the most abundant organic C compounds in the biosphere and are presumably linked to priming effects^62^, we use a low-molecular weight and highly available carbohydrate (glucose) as a trigger-molecule in our priming experimental incubations. Glucose is the most frequently released sugar during rhizodeposition^63^ and a universal substrate for heterotrophic microbes. Given the wide spatial scale of our study, one sole source of a ubiquitous fresh organic matter (glucose) in one conventional dose was utilized. Glucose mineralization never reached 100% (always below 11% of the added glucose-C, Supplementary Fig. 5) in any soil, likely due to the capacity of organo–mineral complexes for stabilizing carbon into the soil^64^. Further, because plants were not used in the microcosms given the large variety of ecosystems, our simplified approach allowed us to remove the natural variation in root exudates and the consequent C inputs. Glucose was applied per soil weight, and not standardized by microbial biomass or SOC content. The reason is that our global survey includes soils with wide ranges in SOC and microbial biomass, but also in many other factors that can regulate the soil priming effect (i.e. clay content, available C content, plant and microbial communities, etc.)^7,17,18,24,34,65^. Thus, unlike in local studies where glucose addition can be standardized, we posit that the most reasonable approach to evaluate a priming effect at the global scale is adding glucose per unit of soil mass weight.

Two parallel sets of 1 g dry soil samples were placed in 20-ml glass vials at 50% of the water-holding capacity, sealed with a rubber septum and pre-incubated for one week at 28 °C in the dark. During this time, microorganisms readapted to the water conditions and released a pulse of CO_2_ due to the new moisture conditions^66^. Similar incubation times were utilized in other priming studies^18,50,67^. Subsequently, glass vials were opened and the atmosphere was refreshed. This standardization was necessary in order to homogenize conditions after the global sampling and storage at −20 °C. After the pre-incubation, glucose mineralization was assayed by adding ^13^C-glucose (99 atom% U-^13^C, Cambridge Isotope Laboratories, Tewksbury, Massachusetts, US) dissolved in water to one of the vial series at a dose of 240 µg of glucose-C per gram of soil. This dose was considerably high but in the range of previous priming studies and affect the growth and structure of the microbial community^14,24,63^. In parallel, the second sample set was subjected to the same procedure adding water without glucose; this sample set was used for measuring basal microbial respiration rates. A total of 172 incubations were conducted in this study (86 soils × two treatments). Then, soils were incubated for 16 days at 28 °C in the dark. Incubations were maintained for more than 2 weeks, because previous studies have revealed that the major part of CO_2_ release from soil tends to occur a few days or weeks after substrate addition and corresponds to apparent priming (microbial biomass turnover)^7^. As reported by some studies focused on soil foodwebs fueled by glucose, this incubation period is sufficient to permit an evolution of microbial populations after glucose addition^49,50^. Longer incubation time was not used as we wanted to avoid CO_2_ saturation in the vials of C-rich soils and because they can incorporate further biases (i.e. nutrient limitations)^68–71^. We are aware that our incubation conditions were outside the range for the mean temperature and water content of soils and, consequently, we estimated the potential apparent priming at the global scale. However, we were interested to know how soil edaphic conditions could influence the direction of apparent priming effects worldwide, and the legacy effects of climate (which would be modified by incubation conditions) are interpreted as indirect effects in our SEM, as discussed below. After incubation, 4 ml of headspace gas from each vial were transferred to pre-evacuated glass vials (Labco Limited, Lampeter, Wales, UK) and the quantity and isotopic composition of released CO_2_ was then determined. The δ^13^C isotope analysis was performed using a Thermo Scientific GasBench-PreCon trace gas system coupled to a Delta V Plus IRMS (Thermo Scientific, Bremen, Germany). The final delta values used for the ^13^C calculations were expressed relative to international standards of V-PDB (Vienna Pee Dee Belemnite^72^). The isotopic ratio of CO_2_ was used to calculate the percentage of CO_2_-C derived from the added glucose or from the soil^73^. Given the short-term nature of the incubation (16 days), the CO_2_ release was interpreted as derived from the microbial biomass turnover, so called apparent priming effect^7–9^. This was defined as the increase or decrease in the CO_2_ derived from the microbial biomass turnover following substrate addition. It was calculated as the total soil respiration following glucose addition minus the amount of C respired from the added ^13^C-glucose and from control soil without glucose amendment^74^; Equation (3)). This was expressed as the extra CO_2_-C (μg) released from soil.

1 $${\mathrm{Priming}}\;{\mathrm{effect}} = \left( {{\mathrm{total}}\;{\mathrm{CO}}_{\mathrm{2}} - {\mathrm{substrate}}\;{\mathrm{derived}}\;{\mathrm{CO}}_{\mathrm{2}}} \right) -{\mathrm{total}}\;{\mathrm{CO}}_{\mathrm{2}}$$

The first component (total CO_2_ – substrate derived CO_2_) refers to the soil amended with substrate and second component (total CO_2_) refers to the unamended soil. Moreover, our metric of priming effect (μg CO_2_-C g^−1^ soil day^−1^) was strongly correlated with priming per unit of soil organic C (μg CO_2_-C g^−1^ soil C day^−1^; *ρ* = 0.82; *p* < 0.001; *n* = 86).

### Microbial biomass and community composition

Microbial biomass was estimated using the substrate-induced respiration approach using Microresp® as described in Campbell et al.^75^. The composition of bacterial and fungal communities was measured via amplicon sequencing using the Illumina MiSeq platform. Ten grams of frozen soil (per sample) were ground using a mortar and liquid nitrogen to homogenize soils and obtain a representative soil sample. Soil DNA was extracted using the Powersoil® DNA Isolation Kit (MoBio Laboratories, Carlsbad, CA, USA) according to the manufacturer’s instructions. A portion of the bacterial 16S (V3–V4 region) and eukaryotic 18S (V9 region) rRNA genes was sequenced using the 341F/805R and Euk1391f/EukBr primer sets, respectively. Bioinformatic processing was performed using a combination of QIIME^76^, USEARCH^77^, and UNOISE3^78^. The relative abundance of microbial phyla was obtained from these analyses. In all, 72/86 samples for fungi and 82/86 samples for bacteria were successfully sequenced and used for statistical analyses below. These samples include soils from all climates and ecosystem types.

### Statistical analyses

First, we first tested for significant differences in priming effect across major ecosystem types using one-way non-parametric Permutational ANalysis Of Variance (PERMANOVA). In these PERMANOVA, each plot is considered a statistical replicate. Put simply, in our study we are using Earth as a grid across which we are collecting data from different plots or sites (replicates) from different ecosystem types. Having more than one sample within each plot would have been considered pseudo-replication as our question was related to comparing the priming effect across different ecosystem types globally (e.g., tropical vs. temperate forests) rather than comparing priming effect across plots within a given ecosystem type (e.g., two temperate forests). Further, gradient designs, as we have used, are powerful tools for detecting patterns in ecological responses to continuous and interacting environmental drivers as they generally outperform replicated designs in terms of prediction success of responses^79^.

Second, we used structural equation modeling (SEM)^32^ to evaluate the direct and indirect relationships between abiotic (pH, salinity, SOC content, soil P content, and texture), biotic (dominant vegetation types, plant cover, respiration rate, SIR-microbial biomass, and relative abundance of bacterial, and fungal phyla) and climatic (MAT and aridity) environmental factors on apparent priming effect based on expectations of an a priori model (Supplementary Fig. 3). Due to the large number of potential microbial taxa predicting soil priming, prior to conducting the SEM, we first used Spearman correlations to identify a negative or positive correlation between the apparent priming and the relative abundance of microbial phyla. Only four taxa were significantly correlated with apparent soil priming (*Armatimonadetes*, *Verrucomicrobia*, *Basidiomycota*, and *Chytridiomycota*; Supplementary Table 1); thus only these taxa were included in our SEM. Of these taxa, we found a significant effect of Basidiomycota only. Our SEM was conducted with the 69 soil samples including matching information for bacterial and fungal community composition. Climate factors (MAT and aridity) are used here as proxies of legacy effects, as incubations for priming effects are done under controlled laboratory conditions, with similar and constant soil water and temperature across all soils^27^. Because of this, we did not include the direct effect of climate on the apparent priming effects and respiration rates. However, we were interested in assessing the indirect effects of climate on priming via changes in SOC content and plant cover, aiming to provide an ecological context to our findings. After attaining a satisfactory model fit, we introduced composite variables into our model. The use of composite variables does not alter the underlying SEM model, but collapses the effects of multiple conceptually related variables into a single composite effect, aiding interpretation of model results. Soil C and basal soil microbial respiration were included as a composite variable, because together they determine the amount of initial SOC content which is respired by microbial communities. Since some of the variables introduced were not normally distributed, the probability that a path coefficient differs from zero was tested using bootstrap tests. Bootstrapping tests do not in such cases assume that the data match a particular theoretical distribution.

### Reporting summary

Further information on research design is available in the Nature Research Reporting Summary linked to this article.

## Supplementary information

Supplementary Information

Reporting Summary

Source Data

## Data Availability

The complete dataset associated with this paper has been deposited in figshare: https://figshare.com/s/56430026ba793775983f (10.6084/m9.figshare.7054265). The source data underlying Fig. 1a–d, and Supplementary Figs. 2 and 5 is available as Source Data file.
