# Supplementary material for: Global ecological predictors of the soil priming effect
Source: Nat Commun. 2019 Aug 2;10:3481. doi: 10.1038/s41467-019-11472-7 (PMC6677791; doi:10.1038/s41467-019-11472-7)
Supplement: Supplementary file 1 — Supplementary Information [file 41467_2019_11472_MOESM1_ESM.pdf]

Supplementary Information

Global ecological predictors of the soil priming effect

Bastida et al.

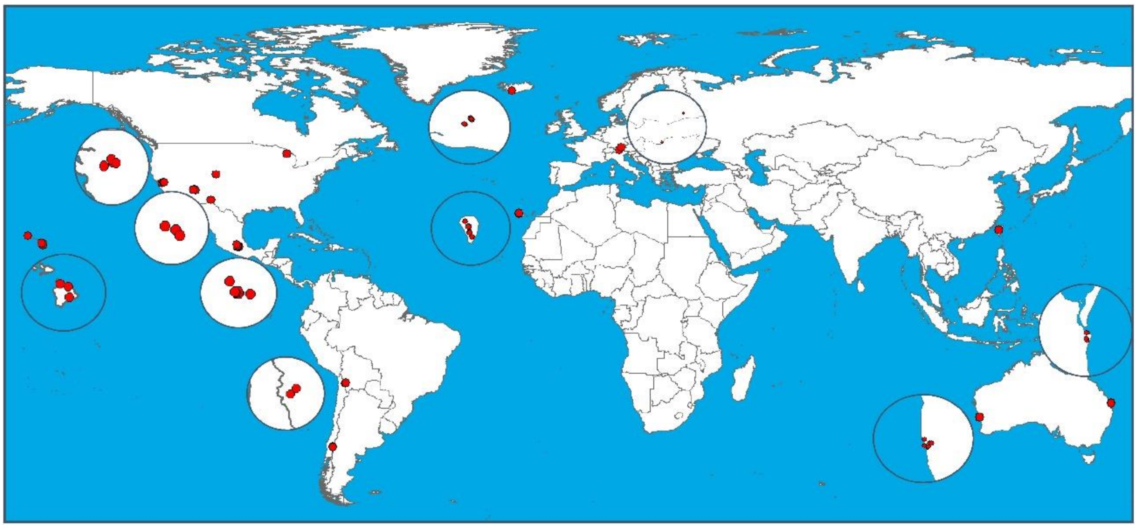

Supplementary Figure 1. Location of the 86 soil samples in this study.

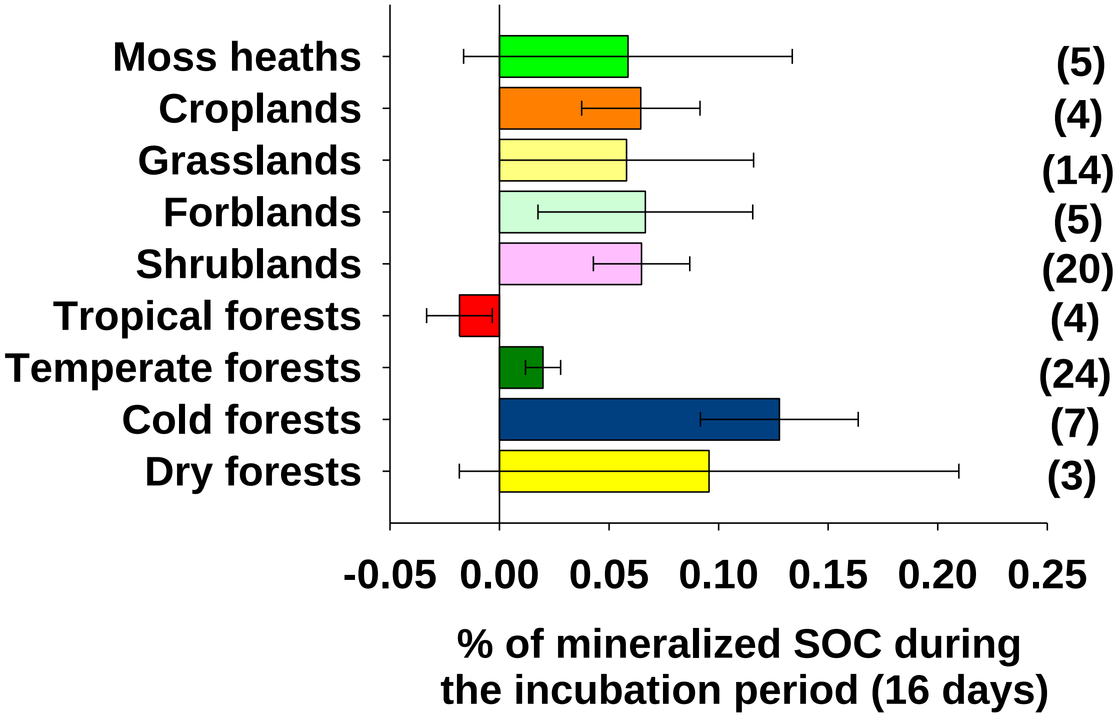

Supplementary Figure 2. Percentage of mineralized soil organic C (SOC) during the incubation period due to apparent priming effects. Number of sites is indicated in parentheses. Error bars are standard error of the mean. Differences among ecosystems were not significant ($p > 0.05$). Source data are provided as a Source Data file.

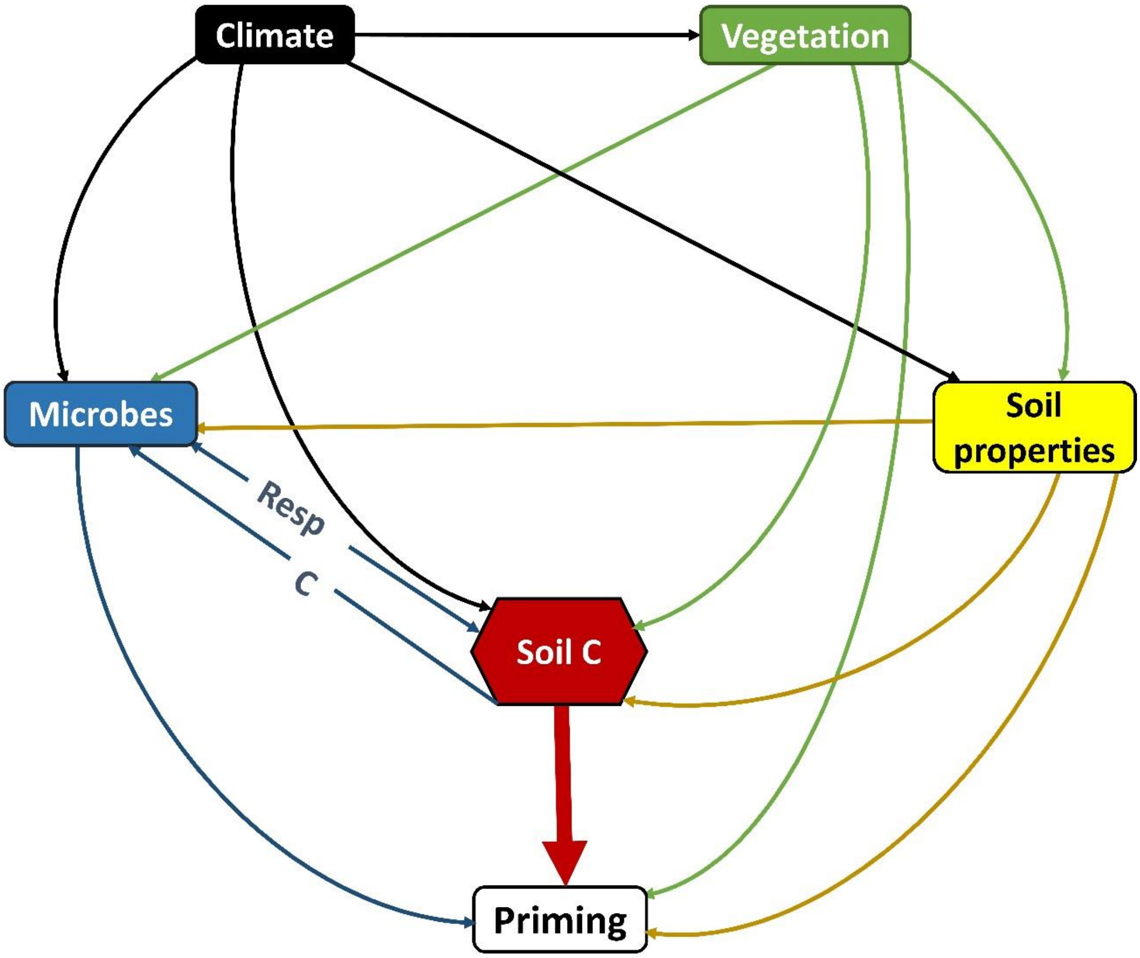

Supplementary Figure 3. *A priori* structural equation modeling (SEM) describing the effects of multiple ecological predictors on the apparent soil priming effect. Climate includes Aridity Index and mean annual temperature (MAT). Vegetation includes presence of forest, grassland and shrubland and plant cover. Soil property includes soil phosphorus (P), salinity, texture and pH. Microbes include the relative abundance of selected microbial taxa and microbial biomass. Soil carbon (C) includes microbial respiration (Resp) and soil organic C.

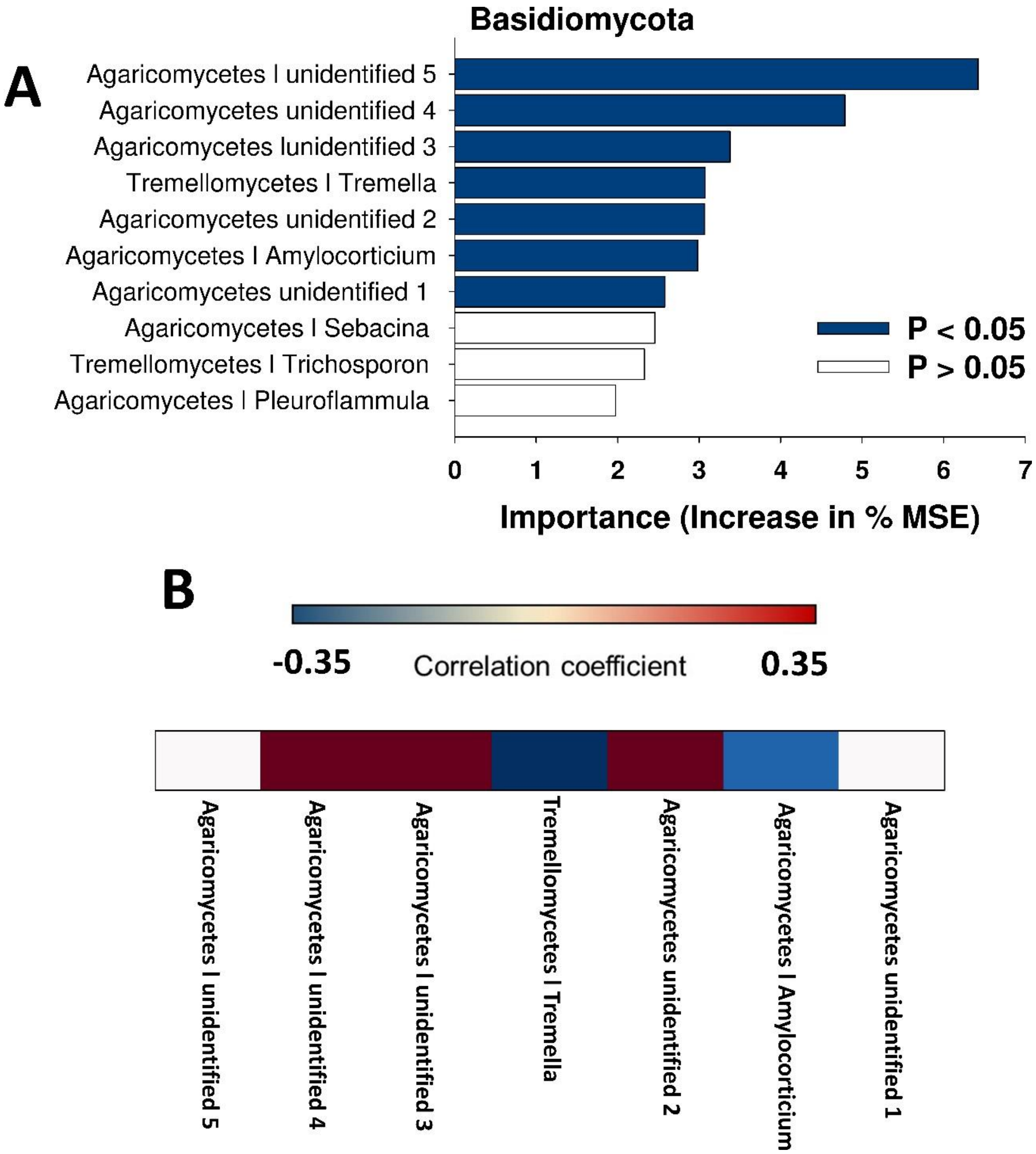

Supplementary Figure 4. Basidiomycota taxa as predictors of apparent priming effect. Panel A includes a Random Forest model identify the top 10 most important taxa predicting the apparent priming effect. Panel B includes the correlation (Spearman; $p < 0.05$) between the relative abundance of taxa selected from Random Forest analyses and the apparent priming effect.

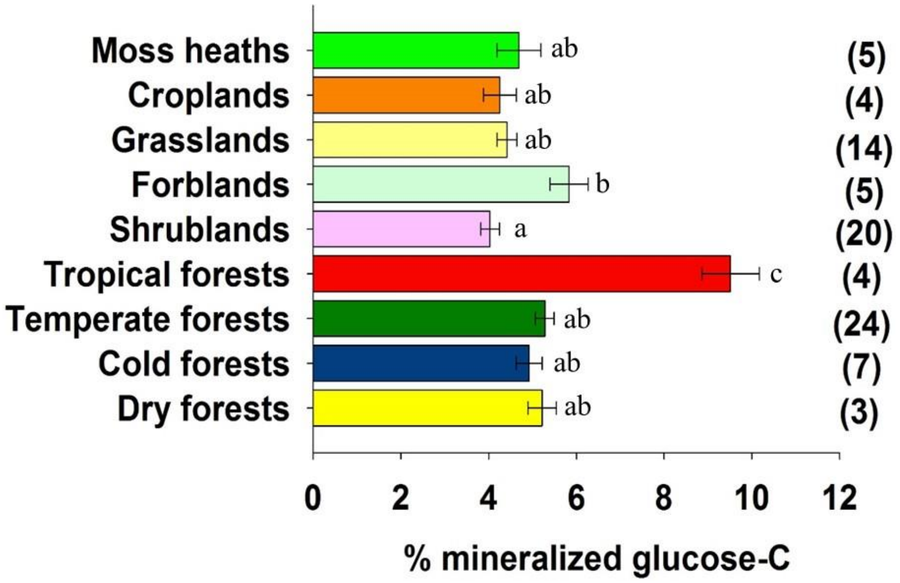

Supplementary Figure 5. Percentage of mineralized glucose-carbon (C) during the incubation period. Number of sites is indicated in parentheses. Error bars are standard error of the mean. Different letters indicate significant differences among ecosystems ($p < 0.001$). Source data are provided as a Source Data file.

Supplementary Table 1. Correlations (Pearson) between the relative abundance of bacterial and fungal taxa and the apparent soil priming effect. Bold text indicates $p < 0.05$.

Microbial group	n	r	*P* value
Acidobacteria	82	-0.184	0.098
Actinobacteria	82	0.151	0.174
AD3	82	0.036	0.751
**Armatimonadetes**	82	**0.228**	**0.040**
Bacteroidetes	82	0.078	0.498
Chlamydiae	82	-0.170	0.128
Chlorobi	82	-0.187	0.093
Chloroflexi	82	0.066	0.556
Cyanobacteria	82	-0.052	0.644
Elusimicrobia	82	-0.201	0.070
Fibrobacteres	82	-0.061	0.587
Firmicutes	82	0.016	0.885
Gemmatimonadetes	82	-0.013	0.905
Nitrospirae	82	-0.004	0.970
Planctomycetes	82	-0.050	0.658
Proteobacteria	82	0.073	0.517
Spirochaetes	82	-0.043	0.700
Tenericutes	82	0.053	0.633
**Verrucomicrobia**	82	**-0.228**	**0.040**
Ascomycota	72	-0.223	0.060
**Basidiomycota**	72	**0.246**	**0.038**
**Chytridiomycota**	72	**-0.299**	**0.011**
Glomeromycota	72	0.094	0.433
Mucoromycota	72	0.025	0.835
