# Supplementary material for: Global ecological predictors of the soil priming effect
Source: Nat Commun. 2019 Aug 2;10:3481. doi: 10.1038/s41467-019-11472-7 (PMC6677791; doi:10.1038/s41467-019-11472-7)
Supplement: Supplementary file 2 — Reporting Summary [file 41467_2019_11472_MOESM2_ESM.pdf]

nature research

Corresponding author(s): Felipe Bastida

Last updated by author(s): May 15, 2019

Reporting Summary

Nature Research wishes to improve the reproducibility of the work that we publish. This form provides structure for consistency and transparency in reporting. For further information on Nature Research policies, see Authors & Referees and the Editorial Policy Checklist.

Statistics

For all statistical analyses, confirm that the following items are present in the figure legend, table legend, main text, or Methods section.

n/a	Confirmed	
☐	☒	The exact sample size (*n*) for each experimental group/condition, given as a discrete number and unit of measurement
☐	☒	A statement on whether measurements were taken from distinct samples or whether the same sample was measured repeatedly
☐	☒	The statistical test(s) used AND whether they are one- or two-sided<br>*Only common tests should be described solely by name; describe more complex techniques in the Methods section.*
☐	☒	A description of all covariates tested
☐	☒	A description of any assumptions or corrections, such as tests of normality and adjustment for multiple comparisons
☐	☒	A full description of the statistical parameters including central tendency (e.g. means) or other basic estimates (e.g. regression coefficient) AND variation (e.g. standard deviation) or associated estimates of uncertainty (e.g. confidence intervals)
☐	☒	For null hypothesis testing, the test statistic (e.g. *F*, *t*, *r*) with confidence intervals, effect sizes, degrees of freedom and *P* value noted<br>*Give P values as exact values whenever suitable.*
☒	☐	For Bayesian analysis, information on the choice of priors and Markov chain Monte Carlo settings
☐	☒	For hierarchical and complex designs, identification of the appropriate level for tests and full reporting of outcomes
☐	☒	Estimates of effect sizes (e.g. Cohen's *d*, Pearson's *r*), indicating how they were calculated

Our web collection on statistics for biologists contains articles on many of the points above.

Software and code

Policy information about availability of computer code

Data collection	No software was used
Data analysis	Data analysis was performed using the R 3.3.2. statistical software

For manuscripts utilizing custom algorithms or software that are central to the research but not yet described in published literature, software must be made available to editors/reviewers. We strongly encourage code deposition in a community repository (e.g. GitHub). See the Nature Research guidelines for submitting code & software for further information.

Data

Policy information about availability of data

All manuscripts must include a data availability statement. This statement should provide the following information, where applicable:

- Accession codes, unique identifiers, or web links for publicly available datasets
- A list of figures that have associated raw data
- A description of any restrictions on data availability

The complete dataset associated with this paper has been deposited in figshare: https://figshare.com/s/56430026ba793775983f (10.6084/m9.figshare.7054265)

Field-specific reporting

Please select the one below that is the best fit for your research. If you are not sure, read the appropriate sections before making your selection.

☐ Life sciences ☐ Behavioural & social sciences ☒ Ecological, evolutionary & environmental sciences

For a reference copy of the document with all sections, see nature.com/documents/nr-reporting-summary-flat.pdf

Ecological, evolutionary & environmental sciences study design

All studies must disclose on these points even when the disclosure is negative.

Study description	we conducted a global survey of soils from 86 locations, spanning six continents and a wide range of climates, vegetation and soil conditions, and evaluated soil priming effects using an isotopic approach.
Research sample	Soil samples from 86 locations across different terrestrial ecosystems in the globe
Sampling strategy	Samples were obtained in six continents and a wide range of climates, vegetation and soil conditions, in order to create a wide range of environmental conditions
Data collection	Data collection was performed by the different authors after sampling and isotope measurements were done in the Stable Isotope Facility, UC Davis, University of California
Timing and spatial scale	Priming was calculated from samples taken in 2016 and 2017 from 86 locations in nine countries and six continents
Data exclusions	We did not exclude any data
Reproducibility	Priming assay, including timing and glucose dose, is clearly described in Methods section and can be reproduced. Isotope calculations are also detailed.
Randomization	Samples were not allocated into experimental groups. They were used to create gradients of different conditions
Blinding	Blinding did not apply to our study as we work with soils and they were not allocated into experimental groups

Did the study involve field work? ☐ Yes ☒ No

Reporting for specific materials, systems and methods

We require information from authors about some types of materials, experimental systems and methods used in many studies. Here, indicate whether each material, system or method listed is relevant to your study. If you are not sure if a list item applies to your research, read the appropriate section before selecting a response.

Materials & experimental systems

n/a	Involved in the study	
☒	☐	Antibodies
☒	☐	Eukaryotic cell lines
☒	☐	Palaeontology
☒	☐	Animals and other organisms
☒	☐	Human research participants
☒	☐	Clinical data

Methods

n/a	Involved in the study	
☒	☐	ChIP-seq
☒	☐	Flow cytometry
☒	☐	MRI-based neuroimaging
